# Predictive value of metabolic activity detected by pre-operative ^18^F FDG PET/CT in ampullary adenocarcinoma

**DOI:** 10.1097/MD.0000000000027561

**Published:** 2021-10-22

**Authors:** Young Mok Park, Hyung Il Seo

**Affiliations:** Division of Hepato-Biliary-Pancreatic Surgery, Department of Surgery, Pusan National University Hospital, Pusan National University School of Medicine, Pusan, Korea.

**Keywords:** ampullary adenocarcinoma, ^18^F-fluorodeoxyglucose positron emission tomography, prognosis, standardized uptake value

## Abstract

In ampullary adenocarcinoma cases, the clinical effects of ^18^F-fluorodeoxyglucose (FDG) positron emission tomography/computed tomography (PET/CT) have not yet been well-studied, unlike other prognostic factors that have been reported till date. This study aimed to investigate the clinical impact of maximum standardized uptake value (SUVmax) in predicting the prognosis of ampullary adenocarcinoma.

Thirty-eight patients who underwent pre-operative ^18^F-FDG PET/CT and curative-intent resection of ampullary adenocarcinoma at Pusan National University Hospital (Pusan, South Korea) between 2008 and 2017 were retrospectively analyzed in this study. We evaluated the clinicopathologic outcomes according to the SUVmax using univariate and multivariate Cox proportional hazard regression analyses and receiver operating characteristic analysis to arrive at a cutoff value.

Lymph node metastasis was detected in 9 patients, and 15 patients experienced a recurrence during the follow-up period. Among 38 patients, 33 showed an increased FDG uptake by the main tumor. SUVmax of 4.55 was selected as a significant independent predictive factor for patient survival along with poor tumor differentiation and high neutrophil-to-lymphocyte ratio in multivariate analysis (*P* = .016, hazard ratio = 5.040). Patients with SUVmax under 4.55 exhibited significantly longer overall survival than the rest (<4.55 vs ≥4.55), and the 5-year overall survival was 82.8% versus 57.4% (*P* = .049).

SUVmax of 4.55 on ^18^F-FDG PET/CT could be a predictive factor for tumor biology and long-term survival in patients with ampullary adenocarcinoma. Nevertheless, considering the cost aspect and its limited prognostic effect, this study seems to require more patient and multicenter studies.

## 1 Introduction

Ampullary adenocarcinoma is a type of periampullary cancer that accounts for 0.2% of gastrointestinal malignancies and 6% of periampullary cancers.^[1,2]^ Ampullary adenocarcinoma has a better prognosis than other periampullary cancers. Compared to other periampullary cancers, ampullary adenocarcinoma tends to be detected relatively early. Therefore, ampullary adenocarcinomas have a higher resection rate at the time of diagnosis. Stage I is most frequently seen in these cancers (21.2%–56.3%), and the overall 5-year survival rate has been reported to be between 40% and 61.3%. The survival rates decrease with increasing pathologic stage.^[3–11]^ Surgical resection is the only potentially curative treatment for patients with ampullary adenocarcinoma. Several prognostic factors, including T category, nodal metastasis, lymphovascular invasion, perineural invasion, blood transfusion, serum carbohydrate antigen 19-9, and loss of body mass index >4%, have been previously reported.^[10–17]^

Currently, ^18^F-fluorodeoxyglucose positron emission tomography/computed tomography (^18^F-FDG PET/CT) is widely used to assess many different types of malignancies, and the maximum standardized uptake value (SUVmax) can be used to predict the survival of patients with malignancy. The extent of the uptake of ^18^F-FDG by cancer that usually correlates with a prognostic impact has shown variable results and is debatable. Recently, a study reported that high SUVmax (>7.5) could be a prognostic factor for overall survival (OS) and disease-free survival (DFS) in ampullary adenocarcinoma.^[18]^ The objective of this study was to assess the prognostic value of pre-operative ^18^F-FDG PET/CT according to metabolic activity and to investigate the clinicopathological differences in this metabolic activity in ampullary adenocarcinoma.

## 2 Patients and methods

### 2.1 Patients

A total of 66 patients who underwent curative resection for ampullary adenocarcinoma at Pusan National University Hospital (Pusan, South Korea) from January 2008 to December 2017 were enrolled in this study. A retrospective review was performed based on medical records. Patients who did not undergo pre-operative ^18^F-FDG PET/CT were excluded, as well as patients with other types of periampullary carcinoma and non-invasive carcinoma (high grade dysplasia or carcinoma in situ, papillary adenocarcinoma). Finally, 38 patients were included. This retrospective study was approved by the institutional review board at Clinical Trial Center (Institutional review board number: 2003-022-089) and written informed consent was obtained from all participants. The clinical information retrospectively reviewed from the patient medical records is shown in Table 1.

**Table 1:** Clinicopathological characteristics of patients with ampullary adenocarcinoma.

Variables	Number of patients, (%
Sex	
Male	21 (55.3)
Female	17 (44.7)
Median age (age range), yrs	64.5 (32–85)
Comorbidity	22 (57.9)
Diabetes	8
Hypertension	13
Cardiovascular disease	4
Dementia	1
Pulmonary disease	2
Other cancer operation	2
CEA (range), ng/mL	2.31 (0.73–24.47)
CA19-9 (range), U/mL	61.01 (0.6–3019)
Albumin (range), mg/dL	3.95 (2.4–4.8)
CRP (range), mg/dL	1.01 (0.05–14.52)
T stage	
T1	14 (36.8)
T2	10 (26.3)
T3	14 (36.8)
Lymph node metastases	
Yes	8 (21.1)
No	30 (78.9)
Tumor size (range), cm	2.2 (0.5–5.5)
Perineural invasion	
Yes	9 (23.7)
No	29 (76.3)
Lymphovascular invasion	
Yes	9 (23.7)
No	29 (76.3)
Differentiation	
Well	10 (26.3)
Moderate	19 (50.0)
Poor	9 (23.7)
Adjuvant treatment	7 (18.4)

### 2.2 ^18^F-FDG PET/CT

All patients underwent fasting for at least 8 hours to ensure a serum glucose level of less than 120 mg/dL. PET/CT imaging was performed 60 minutes after the injection of ^18^F-FDG (5.18 MBq/kg). All scans were performed utilizing one of the 2 systems (Biograph from Siemens Medical Solution, Hoffmann Estates, IL, or Gemini from Philips Medical Systems, Cleveland, OH) using 3-dimensional mode with an acquisition time of 3 minutes per bed position from the base of the skull to the proximal thigh. For a quantitative analysis of the ^18^F-FDG uptake, a region of interest was placed over the most intense area of ^18^F-FDG uptake. The activity concentration within this region was determined and expressed as the standardized uptake value, which was calculated as follows: standardized uptake value = region's radioactivity concentration (Bq/mL)/[injected dose (Bq)/patient's weight (g)].

### 2.3 Statistical analysis

We analyzed clinicopathological features, DFS, and OS rates. OS rate was measured from the date of surgery to the date of death from any cause; locoregional recurrences, distant metastases, and second primary cancer were ignored. DFS rate was measured from the date of surgery to the date of second cancer, locoregional recurrence, distant metastases, or death from any cause. The cutoff value of SUVmax was determined by receiver operating characteristic (ROC) curve analysis. Through ROC curve, the optimal value of SUVmax was identified. SUVmax (4.55) was selected as optimal cutoff value for quantitative SUVmax. The SUVmax from ^18^F-FDG PET/CT and other tumor factors were compared between the following 2 subgroups using the chi-square test: High metabolism group (SUVmax ≥ 4.55) and low metabolism group (SUVmax < 4.55). The chi-square test was used to compare all categorical variables. OS and DFS were estimated according to the Kaplan-Meier method, and survival differences were evaluated using the log-rank test. Both univariate and multivariate Cox proportional hazard regression models were used to identify risk factors for recurrence or death. Risk factors obtained from univariate models were included in the multivariate models. All statistical analyses were performed using SPSS software (version 20.0; SPSS Inc, Chicago, IL), and *P* values < .05 were considered statistically significant.

## 3 Results

### 3.1 Patient characteristics

The study cohort consisted of 21 men and 17 women. The median age was 64 years (range, 32–85). There were 24 patients with T1 and T2 tumors (63.1%), and lymph node metastases were detected in 8 patients. The median number of retrieved lymph node was 20.7 (range, 4–67). According to the 7th edition of the cancer staging manual by American Joint Committee on Cancer, 14 patients (36.8%) were classified as Stage IA, 7 (18.4%) were classified as Stage IB, 9 (23.7%) were classified Stage IIA, and 8 (21.1%) classified as Stage IIB. Adjuvant treatment was administered to 7 patients (5 received concomitant chemoradiation therapy and 2 received only chemotherapy) with lymph node metastases (Table 1).

### 3.2 SUVmax according to patient clinicopathological characteristics

**Figure 1:**
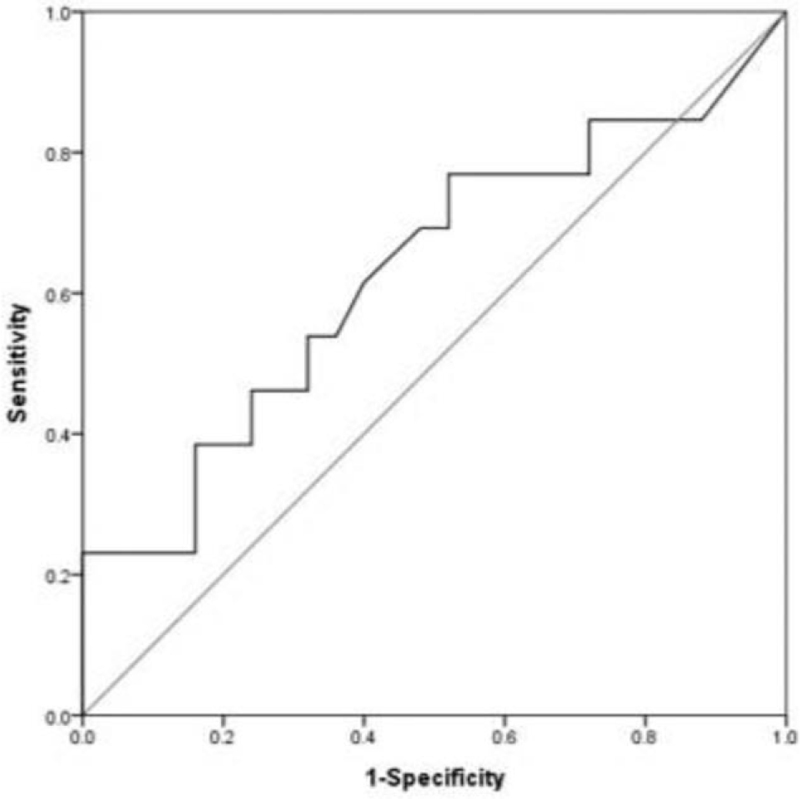
Receiver operating characteristic curve of maximum standardized uptake value distribution with 4.55 as cutoff value, 61.5% sensitivity, and 60.0% specificity.

**Figure 2:**
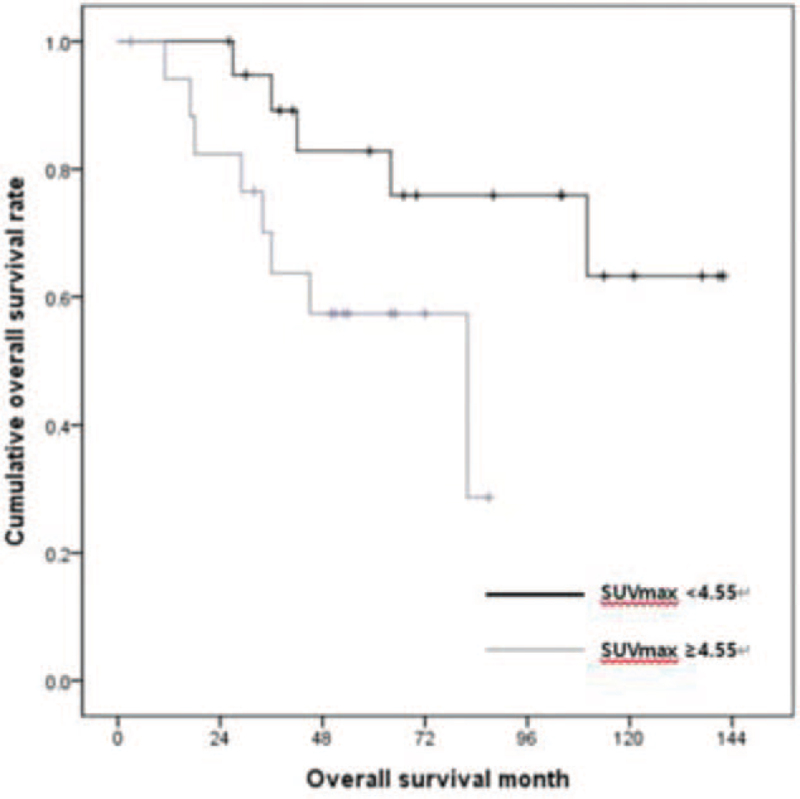
Overall survival according to the metabolic activity based on maximum standardized uptake value of 4.55 and *P* = .05. SUVmax = maximum standardized uptake value.

The cutoff value of SUVmax was determined by an ROC curve analysis. According to the ROC curve, the SUVmax cutoff value for patient's survival was 4.55, and the sensitivity and specificity were 61.5% and 60.0% (95% confidence intervals: 0.431–0.828), respectively (Fig. 1). For disease recurrence, there were no statistical significances of SUVmax. OS difference was observed with an SUVmax cutoff value of 4.55, and the 1-, 3-, and 5-year OS rates based on an SUVmax of 4.55 in the 2 groups were 100%, 89.2%, and 82.8%, respectively versus 94.1%, 63.7%, and 57.4%, respectively. Patients with SUVmax < 4.55 had a significantly longer survival (*P* = .049) (Fig. 2). Patient characteristics according to metabolic activity are shown in Table 2. Patients with SUVmax ≥ 4.55 had higher platelet-neutrophil-lymphocyte (PNL) ratio (*P* = .05), larger tumor size (*P* = .02), more advanced T stage (*P* = .02), lymph node metastases (*P* = .01), and lymphovascular invasion (*P* = .04). PNL ratio, tumor size, T state, nodal status, and lymphovascular invasion are well-known significant prognostic factors for periampullary adenocarcinoma.

**Table 2:** Comparison of patient characteristics according to metabolic activity.

Characteristic	No.	SUVmax < 4.55 n = 20, (%)	SUVmax ≥ 4.55 n = 18, (%)	*P* value
Age, yrs				.94
<65	19	10 (50.0)	9 (50.0)	
≥65	19	10 (50.0)	9 (50.0)	
Sex				.18
Male	21	9 (45.0)	12 (66.7)	
Female	17	11 (55.0)	6 (33.3)	
CEA, ng/mL				.17
<5	28	15 (93.8)	13 (76.5)	
≥5	5	1 (20.0)	4 (27.8)	
CA19-9, U/mL				.24
<39	15	9 (52.9)	6 (33.3)	
≥39	20	8 (47.1)	12 (66.7)	
Albumin, mg/dL				.19
<3.5	6	4 (52.9)	2 (11.1)	
≥3.5	32	16 (47.1)	16 (88.9)	
CRP, mg/dL				1.00
<1.0	19	10 (50.0)	9 (50.0)	
≥1.0	19	10 (50.0)	9 (50.0)	
GPS				.73
0	18	10 (50.0)	8 (44.4)	
1–2	20	10 (50.0)	10 (55.6)	
mGPS				1.00
0	19	10 (50.0)	9 (50.0)	
1–2	19	10 (50.0)	9 (50.0)	
PL ratio				.52
<163	19	11 (55.0)	8 (44.4)	
≥163	19	9 (45.0)	10 (55.6)	
NL ratio				.52
<3.5	22	13 (65.0)	9 (50.0)	
≥3.5	16	7 (35.0)	9 (50.0)	
PNL ratio				.05
<951	21	14 (70.0)	6 (38.9)	
≥951	17	7 (30.0)	12 (61.1)	
T status				.02
1–2	24	16 (80.0)	8 (44.4)	
3	14	4 (20.0)	10 (55.6)	
Tumor size (cm)				.02
<2.2	18	13 (65.0)	5 (27.8)	
>2.2	20	7 (35.0)	13 (72.2)	
Node metastasis				.01
No	30	19 (95.0)	11 (61.1)	
Yes	8	1 (5.0)	7 (38.9)	
Perineural invasion				.18
No	29	17 (85.0)	12 (66.7)	
Yes	9	3 (15.0)	6 (33.3)	
Lymphovascular invasion				.04
No	29	18 (90.0)	11 (61.1)	
Yes	9	2 (10.0)	7 (38.9)	
Histologic differentiation				.57
Well to moderate	29	16 (80.0)	13 (72.2)	
Poor	9	4 (20.0)	5 (27.8)	

### 3.3 Patient survival and disease recurrence

**Figure 3:**
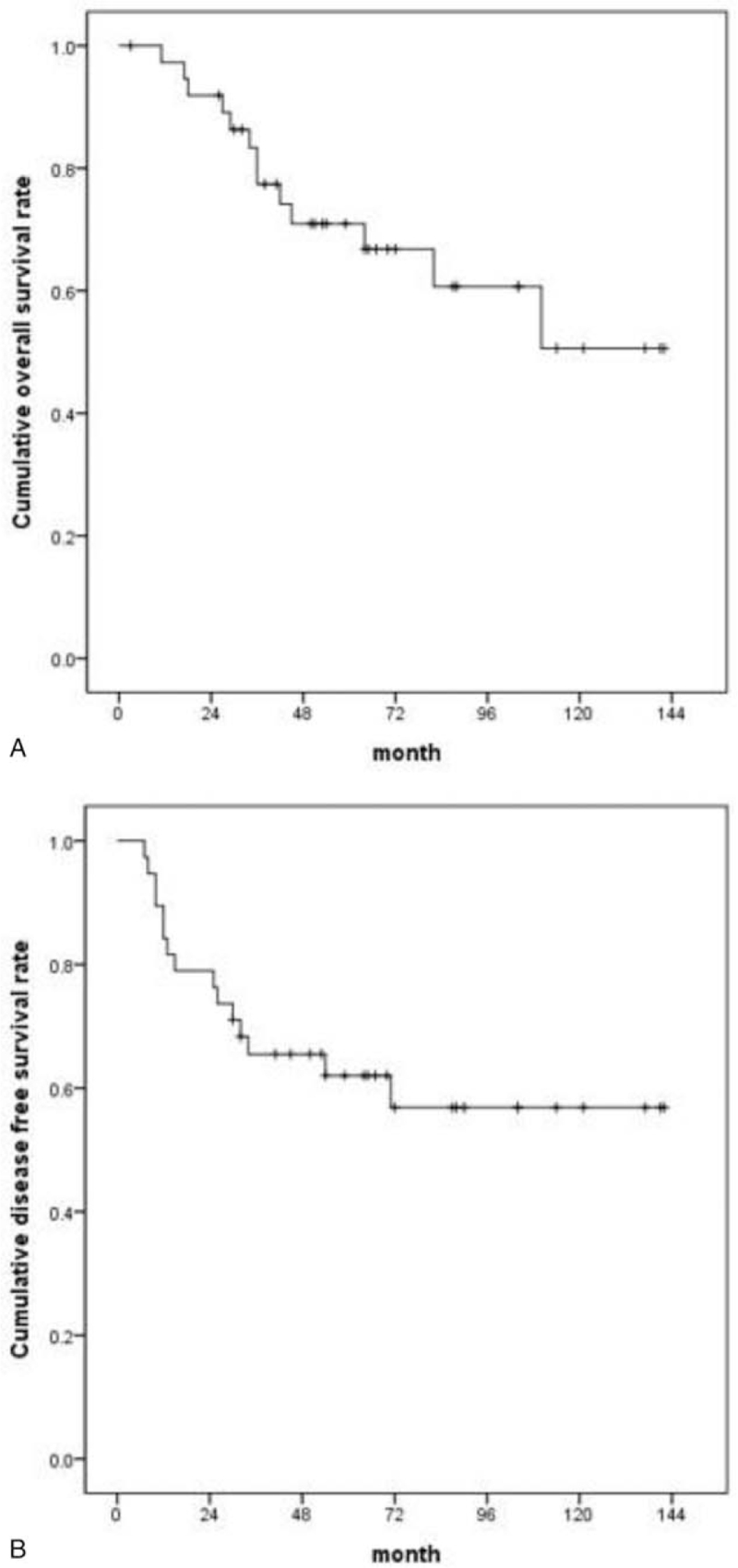
(A) Overall survival rates for 1-year, 3-year, and 5-year were 97.3%, 83.3%, and 70.9%, respectively, and (B) disease-free survival rates for 1-year, 3-year, and 5-year were 89.5%, 65.5%, and 62%, respectively.

Of the 38 patients enrolled in this study, 15 patients experienced a recurrence (4 had locoregional, 4 liver, 3 lung, 3 peritoneum, and 1 bone) during the clinical follow-up period after curative resection. One patient died without a recurrence. The median duration of follow-up after surgery was 54 months. The 5-year OS rate after surgery was 70.9% (Fig. 3A), and the 5-year DFS rate was 62.0% (Fig. 3B).

We performed univariate analysis to evaluate the relationship between the clinicopathological variables and OS. This analysis revealed that patient survival was associated with histologic differentiation, T stage, nodal status, lymphovascular invasion, platelet-to-lymphocyte (PL) ratio, neutrophil-to-lymphocyte (NL) ratio, and PNL ratio. Among these factors, the cutoff level of quantitative values such as PL, NL, and PNL ratio were determined by a ROC curve analysis. According to the ROC curve, cutoff value, the sensitivity and specificity of PL, NL, and PNL ratio were PL (163, 76.9% and 64.0%), NL (3.5, 76.9% and 72.0%), and PNL (951, 76.9% and 72.0%). In the subsequent multivariate analysis, we found that poor histologic differentiation, high SUVmax, and NL ratio were independent risk factors for patient survival (*P* = .004, hazard ratio [HR] = 6.560, *P* = .016, HR = 5.040 and *P* = .003, HR = 7.658, respectively) (Table 3).

**Table 3:** Analysis of predictive factors for overall and disease-free survival.

	Overall survival				Disease-free survival			
	Univariate	Multivariate			Univariate	Multivariate		
Variables	*P*	*P*	HR	95% CI	*P*	*P*	HR	95% CI
Age (≥65 yrs)	.61	–	–	–	.91	–	–	–
Sex	.70	–	–	–	.58	–	–	–
Co-morbidities	.39	–	–	–	.44	–	–	–
Pre-operative biliary drainage	.41	–	–	–	.60	–	–	–
CEA (≥5, ng/mL)	.92	–	–	–	.44	–	–	–
CA19-9 (≥39, U/mL)	.32	–	–	–	.41	–	–	–
Albumin (<3.5, mg/dL)	.83	–	–	–	.79	–	–	–
CRP (≥1, mg/dL)	.38	–	–	–	.14	–	–	–
Tumor differentiation (Poor)	.009	.004	6.560	1.802–23.86	.002	.027	3.328	1.144–9.678
T stage (≥3)	.009	.25	0.370	0.069–1.980	.01	.21	0.459	0.137–1.533
Tumor size (≥2.2, cm)	.18	–	–	–	.69	–	–	–
Node metastasis	.004	.36	0.371	0.045–3.049	.001	.45	0.541	0.109–2.697
Lymphovascular invasion	.004	.33	0.331	0.035–3.118	.001	.85	0.849	0.156–4.620
Perineural invasion	.73	–	–	–	.23	–	–	–
GPS (≥1)	.17	–	–	–	.06	–	–	–
mGPS (≥1)	.38	–	–	–	.14	–	–	–
SUVmax (≥4.55)	.049	.016	5.040	1.359–18.69	.48	–	–	–
PL ratio (≥163)	.04	.50	0.371	0.021–6.540	.13	–	–	–
NL ratio (≥3.5)	.002	.003	7.658	1.967–29.82	.002	.018	4.245	1.279–14.09
PNL ratio (≥951)	.002	.62	0.544	0.050–5.911	.004	.50	0.563	0.106–2.989

We also performed univariate analysis to evaluate the relationship between the clinicopathological variables and disease recurrence. This revealed that DFS was associated with histologic differentiation, T stage, nodal status, lymphovascular invasion, NL ratio, and PNL ratio. In the multivariate analysis, poor histologic differentiation and NL ratio were the statistically significant risk factors for disease recurrence (*P* = .027, HR = 3.328, *P* = .018, HR = 4.245, respectively) (Table 3).

### 3.4 Clinical course of PET-negative patients

PET negative findings in periampullary adenocarcinoma has been known as a good prognostic factor, but there may be some cases that give different results than expected. Table 3 showed that among 38 patients, 5 patients exhibited no FDG uptake (PET-negative) in the cancer lesion on ^18^F-FDG PET/CT. The 5-year OS rate was 80.0%, and the 5-year DFS rate was 80% in these PET-negative patients. There was no survival difference between PET-negative and PET-positive patients. Among 5 patients, only 2 cases of death were seen. The case 4 with bone metastasis had several poor prognostic factors such as poor tumor differentiation, high NL. The patient received radiotherapy for bone metastasis but died because of sepsis after 2 months. But the case 3 patient did not have another factors that could make the prognosis worse. She was found to have lymph node metastases in Roux-en-Y jejunal limb and received multimodal treatments, including palliative concomitant chemoradiation therapy, palliative chemotherapy, and radiofrequency ablation. She died because of aggravating liver and lung metastases at 111 months postoperatively (Table 3).

## 4 Discussion

In the present study, we evaluated the significance of SUVmax measured by pre-operative ^18^F-FDG PET/CT for predicting the prognosis of patients with ampullary adenocarcinoma. Our results demonstrate that SUVmax of 4.55 on ^18^F-FDG PET/CT could be a predictive factor for tumor biology and long-term survival in patients with ampullary adenocarcinoma.

Although better than other periampullary adenocarcinomas, the prognosis of ampullary adenocarcinoma still remains poor. Recurrence rate approaches 40% and 5-year OS ranges from 33% to 68%. Our study results were concurrent with the previous reports: the disease recurrence rate was 39%, and the 5-year OS and DFS rates were 70.9% and 62.0%, respectively.^[19,20]^

There have been reports about various poor prognostic factors, including T category, nodal metastasis, lymphovascular invasion, perineural invasion, blood transfusion, and serum carbohydrate antigen 19-9. Furthermore, systemic inflammatory response has been proven to be closely associated with cancer initiation, promotion, malignant conversion, invasion, and metastasis. Several inflammatory biomarkers, including C-reactive protein (CRP), albumin, PL ratio, NL ratio, and PNL ratio, have been reported using pre-operative blood testing. Cutoff values remain unknown, although all studies agree that a high titer of these biomarkers reflects poor prognosis.^[21–25]^

NL ratio has been reported as a predictor of prognosis in patients with several types of digestive tract cancers, including esophageal, gastric, colorectal, pancreatic and gallbladder cancer, cholangiocarcinoma, liver metastasis from colorectal cancer, and hepatocellular carcinoma. Haruki et al^[26]^ reported that routine pre-operative NL ratio measurement in patients undergoing curative treatment for ampullary adenocarcinoma may provide a means of identifying patients with poorer prognosis. They demonstrated that a NL ratio >3 was an independent and significant predictor of poor OS.^[27]^

In our study, poor histologic differentiation, high SUVmax, and NL ratio were significant predictive factors associated with patient survival, and poor histologic differentiation and NL ratio were significant predictive factors associated with disease recurrence. NL ratio was an independent predictive factor for both OS and DFS.

Although variations in the uptake of FDG are known to exist among tumor types, an elevated uptake of FDG has been demonstrated in most primary malignancies.^[28,29]^^18^F-FDG PET/CT has been widely used for not only diagnosis of malignancy but also cancer staging, detection of recurrence, and monitoring of treatment. However, in ampullary adenocarcinoma, the clinical effects of ^18^F-FDG PET/CT have not yet been well-studied, unlike other prognostic factors that have been reported so far. To date, a few studies have reported the clinical usefulness of ^18^F-FDG PET/CT in the detection and characterization of primary tumor, pre-operative staging, detection of recurrence disease, and response to chemotherapy. Choi et al^[30]^ reported that high SUVmax (>4.8) was associated with poor survival outcomes.

In our study, SUVmax (≥4.55) was a significant predictive factor of poor survival. Moreover, we demonstrated that the high metabolism group (SUVmax ≥ 4.55) showed significant correlation with advanced T stage, larger size, lymph node metastasis, lymphovascular invasion, and high PNL ratio. We can assume that these factors influence the outcomes of high metabolic PET/CT activity, which may play an important role in assessing the prognosis of periampullary adenocarcinoma.

Detection of ^18^F-FDG on PET/CT depends on both the size of the lesion and the degree of uptake, as well as surrounding background uptake and intrinsic resolution of imaging. CRP and hyperglycemia have been reported to be highly associated with detectability.^[31]^ Iwano et al^[32]^ reported that in general, tumor lesions ≤2 cm in diameter and well-differentiated carcinomas on thin-section CT images have a tendency toward negative findings on PET scans.

All patients with negative PET findings in our study had small sized tumor below 2.2 cm, lower tumor stage, no node metastasis, and negative lymphovascular invasion. In only 1 case among these patients, the disease recurred despite the absence of poor tumor biology. Another patient with poor tumor biology (poor differentiation and high NL/PNL ratio) showed disease recurrence and eventually died. However, in this case, uneven high levels of CRP were observed. Therefore, small-sized tumor, high level of CRP, and good tumor biology may be the causes of false-negative PET findings (Table 4).

**Table 4:** Clinicopathological features of positron emission tomography negative patients.

Case	Sex/age (yrs)	Size (cm)	CRP (mg/dL)	Tumor differentiation	TNM stage	Lymphovascular invasion	NL ratio (≥3.5)	Recurrence (mos)	Result (mos)
1	F/68	1.5	0.24	Moderate	T2N0	–	–	NED	Alive (144)
2	M/61	1.5	1.26	Moderate	T1N0	–	+	NED	Alive (144)
3	F/60	1.2	0.42	Well	T1N0	–	–	Locoregional (60)	Dead (111)
4	F/85	2.1	9.16	Poor	T2N0	–	+	Bone (26)	Dead (28)
5	F/65	2.0	0.36	Well	T1N0	–	–	NED	Alive (72)

Curative resection is the best option for ampullary adenocarcinoma, but its survival benefit is still compromised by tumor recurrence. Although the efficacy of neoadjuvant treatment has not been proven yet, we can assume that PET/CT may be helpful to select the patients for such a treatment.

There were some differences between our study and previous reports, which might be due to the limitations of this study. The limitations of this study are associated with its retrospective nature, high incidence of T1, 2 stages, and small number of cases with ampullary adenocarcinoma. In addition, in this study, the SUVmax of 4.55 showed marginal significance, which cannot be excluded from the possibility of interference with a known poor prognostic factor. This is largely due to the absolute lack of experimental group for PET/CT. Increasing the size of the target group is expected to significantly improve the limitations of the current study caused by low sensitivity and specificity. The current policy of the insurance application of PET/CT in the domestic reality seems to act as a big factor that interfere to proceed such research effectively and reliably. Therefore, we believe that if these problems are improved in the future, more reliable research results will be derived.

In conclusion, we demonstrated that poor histologic differentiation, high SUVmax, and NL ratio were significant poor prognostic factors for patient survival. Additionally, poor differentiation and NL ratio were also significant prognostic factors for DFS in ampullary adenocarcinoma. We also showed that SUVmax of 4.55 on ^18^F-FDG PET/CT could be a predictive factor for tumor biology and long-term survival in patients with ampullary adenocarcinoma. Nevertheless, considering the procedure cost and limited prognostic effect, further research of this method using larger cohorts and multicenter studies is warranted.

## Author contributions

**Conceptualization:** Hyung Il Seo, Young Mok Park.

**Data curation:** Hyung Il Seo, Young Mok Park.

**Formal analysis:** Hyung Il Seo, Young Mok Park.

**Funding acquisition:** Hyung Il Seo, Young Mok Park.

**Investigation:** Hyung Il Seo, Young Mok Park.

**Methodology:** Hyung Il Seo, Young Mok Park.

**Project administration:** Hyung Il Seo, Young Mok Park.

**Resources:** Hyung Il Seo, Young Mok Park.

**Software:** Hyung Il Seo, Young Mok Park.

**Supervision:** Hyung Il Seo, Young Mok Park.

**Validation:** Hyung Il Seo, Young Mok Park.

**Visualization:** Hyung Il Seo, Young Mok Park.

**Writing – original draft:** Young Mok Park.

**Writing – review & editing:** Young Mok Park.
